# Posterior Fontanelle Encephalomeningocele in a Neonate: A Case Report

**DOI:** 10.7759/cureus.2315

**Published:** 2018-03-13

**Authors:** Abdurrahman Raeiq

**Affiliations:** 1 Neurosurgery, Sir Charles Gairdner Hospital, Perth, Australia

**Keywords:** encephalocele, meningocele, encephalomeningocele, neonate, posterior fontanelle, instrumented delivery, forceps delivery

## Abstract

Encephalomeningoceles are subtypes of neural tube defects (NTD). We present the case of a one-day-old neonate who was found to have a posterior fontanelle encephalomeningocele that was only discovered after birth. The unique presentation of this case and the surgical management is also considered.

## Introduction

Neural tube defects (NTD) are developmental abnormalities of the neural tube, the most common type being spina bifida. Since the widespread public health initiative of introducing folic acid supplementation, NTDs have decreased in prevalence [1]. Encephalomeningoceles are another type of NTD, less common than spina bifida. It involves sac-like protrusions of the brain and surrounding membranes through defects in the skull. The name reflects the fact that the protrusions can involve the membrane, membrane and brain, or rarely, just the brain. Large defects are often discovered in prenatal ultrasound screening. Failing prenatal detection, the defects are discovered at birth by direct sight. Rarely is there a delay after birth in identifying cases. We present a case of a newborn child that was found to have encephalomeningocele one hour after birth.

## Case presentation

A newborn male child, less than one day old, was found to be leaking clear fluid from the skull one hour after birth whilst the mother was holding the child. The child was born at forty-one weeks through a normal vaginal delivery with forceps assistance and no immediate complications. Prenatal screening was unremarkable. The mother was from an indigenous background and in good health and there was no family history of any other similar illnesses. She denied smoking and alcohol use during pregnancy. At birth, no issue with the skull was noted. On further inspection of the child, after the mother raised concerns about the clear fluid leaking from the skull, a 1.5 cm circular defect was identified in the area of the posterior fontanelle with macerated skin edges and underlying membranes and evidence of clear fluid flowing from it. The child had normal haemodynamic parameters throughout and was otherwise examined normally. Further testing of the fluid for beta-trace protein was positive for cerebrospinal fluid (CSF). The neurosurgery team at the tertiary referral centre was contacted at this stage for a suspected encephalomeningocele and the child was transferred there for further management. Further examination at this stage showed a child who was otherwise well, feeding normally, and with normal system review.

**Figure 1:**
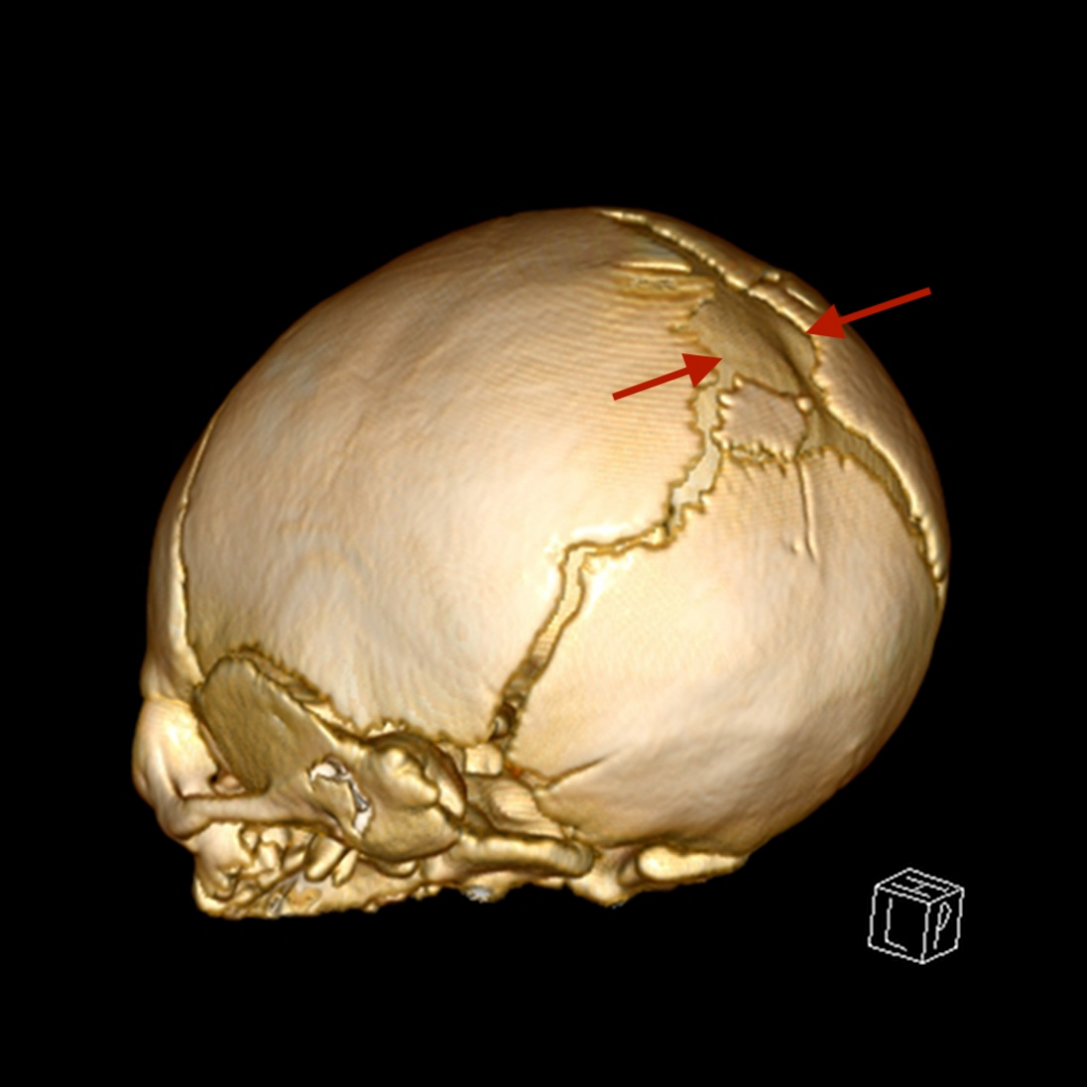
3D-reconstructed skull view showing a posterior defect in between the red arrows

**Figure 2:**
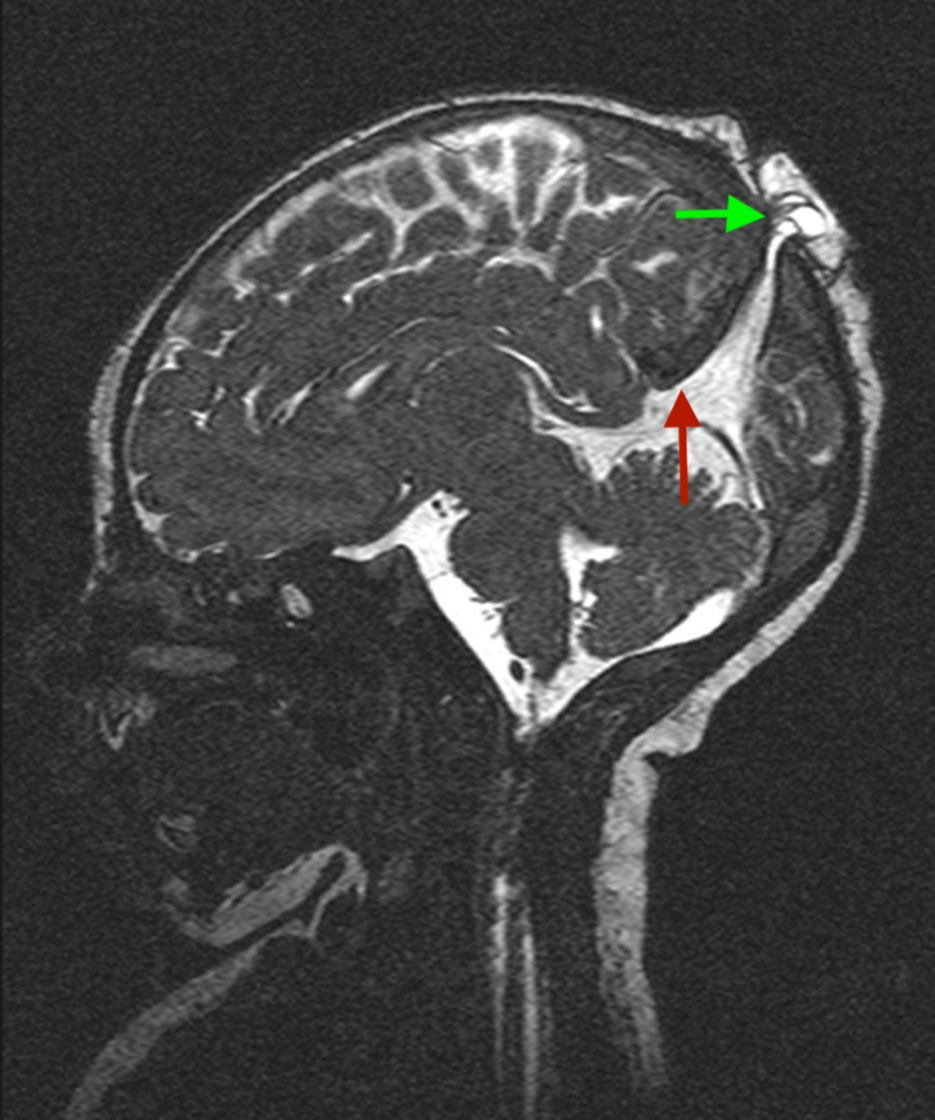
Sagittal CISS T2-enhanced brain MRI showing encephalomeningocele and associated membranes (green arrow), and persistent fetal vein (red arrow) CISS: constructive interference in steady state; MRI: magnetic resonance imaging.

Further imaging with computed tomography (CT) scan of the head, magnetic resonance imaging (MRI) of the brain, magnetic resonance angiography (MRA), and magnetic resonance venography (MRV) identified a likely encephalomeningocele (Figures 1-2). The defect was arising from the posterior fontanelle. Characterisation of the surrounding vasculature showed a persistent fetal vein and possibly an associated vein within the defect. An MRI spine to identify any associated NTD was unremarkable.

The child was booked in for emergent repair of the encephalocele. During the operation, a vertical incision with an elliptical extension around the defect was marked. Careful dissection from superior and inferior aspects of the incision to the pericranium and then working towards the defect identified the defect which appeared to consist of meninges. There were adjacent large venous structures visible beneath the defect and superiorly the superior sagittal sinus was identified in the midline. The defect was dissected from a peripheral direction towards a centre where the herniation appeared to arise from. At this stage, a small pedicle of tissue was holding the structure at the centre. This was tied off with absorbable sutures as there was a high possibility of vascular structures within the pedicle. Bipolar cautery was used to separate the defect. No obvious vessel injury was identified. A pericranial flap was mobilised inferiorly and was reflected around its pedicle from below and over the defect and sutured in place beyond the superior aspect of the defect to pericranium. A piece of synthetic dural graft material was secured over this repair. The wound was closed in two layers with braided absorbable sutures to the galea and then unbraided absorbable sutures to the skin. No further leak was identified at the conclusion of the operation. There was no obvious brain tissue on view during surgery, subsequent pathology also confirmed that no brain tissue was included in the specimen sent. This would support a diagnosis of a meningocele as only meninges were involved rather than an encephalomeningocele. The patient returned to the neonatal intensive care unit postoperatively and was extubated without issue. The child made an excellent recovery and was discharged six days after surgery.

## Discussion

In countries that hold accurate records, NTDs have shown decreased prevalence [1]. Defects have been associated with folate deficiency and supplementation has been responsible for the decreased prevalence by up to 70% [2]. Prenatal ultrasound can identify most defects; however, it is plausible that smaller defects can be missed during maternal screening [3]. Folate deficiency does not explain all cases of NTD and it has been suggested that up to 70% of cases are related to genetic causes [4].

Most are managed conservatively and repaired in a delayed manner, within months to a few years later, to minimise surgical morbidity and mortality. CSF leak, in this case, was an indication of early repair due to the risk of meningitis. This case was unusual in that the defect and associated leak was identified an hour after birth. This may have been due to a spontaneous rupture and leaking, or failure of the delivery suite in examining the child thoroughly enough to miss an obvious defect. The child was delivered with forceps assistance; however, there is no clear indication what role this played in this instance. It is possible that instrumentation, in this case, could have caused a puncture of the herniated meninges but there is a lack of evidence to support this. Review of the literature failed to identify any similar cases of a meningocele, in the context of a forceps delivery presenting with a delayed CSF leak. Work up should include careful assessment of the bony structures as well as identification of often-associated vascular anomalies. Persistent fetal veins can be present [5]. In addition, venous structures can present a real danger during surgery with significant blood loss that can be fatal in a such a young child with its low circulating blood volume.

Operative management can include many approaches, and careful workup and meticulous technique can decrease the risk of adverse outcome. Due to the great healing potential of the young child and the fusion of the cranial bones as a part of normal development, skull defects often heal very well. The long-term prognosis is usually excellent in cases with small defects and where imaging identifies normal brain development. Larger defects and/or those associated with significant underlying brain development abnormalities often carry worse outcomes [4].

## Conclusions

This case highlights a rare type of defect still seen in the developed world. It is evident that careful skull examination of the newborn should be carried out at birth to identify such defects and care should be exercised in using instrument-assisted delivery. The child was investigated with MRI brain, CT head, MRV, MRA and MRI spine. Surgery was planned to avoid potential large vascular structures. An emergent repair was carried out due to the presence of an ongoing CSF leak and the risk of meningitis that this represented. A simple repair technique was presented to manage such a condition and the child made an uneventful recovery and was discharged home to the care of his parents.
